# *Mycobacterium tuberculosis* extracellular vesicle-associated lipoprotein LpqH as a potential biomarker to distinguish paratuberculosis infection or vaccination from tuberculosis infection

**DOI:** 10.1186/s12917-019-1941-6

**Published:** 2019-06-07

**Authors:** Ainhoa Palacios, Leticia Sampedro, Iker A. Sevilla, Elena Molina, David Gil, Mikel Azkargorta, Felix Elortza, Joseba M. Garrido, Juan Anguita, Rafael Prados-Rosales

**Affiliations:** 1 CIC bioGUNE, Bizkaia Technology Park, Building 801A, 48160 Derio, Bizkaia Spain; 2 NEIKER-Instituto Vasco de Investigación y Desarrollo Agrario, Animal Health Department, Bizkaia Science and Technology Park, Derio, Bizkaia Spain; 3 Department Microbiology and Immunology, Albert Einstein College of Medicine, NY, Bronx, USA; 4 Electron microscopy platform, CIC bioGUNE, Bizkaia Technology Park, Derio, Bizkaia Spain; 5 Proteomics platform, CIC bioGUNE, Bizkaia Technology Park, Derio, Bizkaia Spain; 6 Ikerbasque, Basque Foundation for Science, Bilbao, Bizkaia Spain; 7 Department of Preventive Medicine, Public Health and Microbiology, Faculty of Medicine, Autonoma University of Madrid, Madrid, Spain

**Keywords:** Extracellular vesicles, *Mycobacterium tuberculosis*, *Mycobacterium avium paratuberculosis*, *Mycobacterium bovis*, Plasma, LpqH, Lipoprotein

## Abstract

**Background:**

Both bovine tuberculosis (TB) and paratuberculosis (PTB) are serious and widespread bacterial infections affecting many domestic and wild animal species. However, current vaccines do not confer complete protection and cause interference with other diagnostics tests, including bovine TB. Therefore, the development of “Differentiating Infected from Vaccinated Animals” (DIVA) tests are a pressing need. In this study, we have tested the feasibility of mycobacterial extracellular vesicles (EVs) as potential source of biomarkers to discriminate between *Mycobacterium bovis* infected, *Mycobacterium avium subsp. paratuberculosis* (MAP) infected and MAP-vaccinated cows. We have, initially, characterized vesicle production in the two most medically relevant species of mycobacteria for livestock, MAP and *M. bovis*, for being responsible for tuberculosis (TB) and paratuberculosis (PTB).

**Results:**

Our results indicate that these two species produce EVs with different kinetics, morphology and size distribution. Analysis of the immunogenicity of both type of EVs showed some cross reactivity with sera from PTB+ and TB+ cows, suggesting a limited diagnostic capacity for both EVs. Conversely, we noticed that *Mycobacterium tuberculosis* (Mtb) EVs showed some differential reactivity between sera from MAP-vaccinated or PTB+ cows from TB+ ones. Mass spectrometry analysis (MS) identified a 19-kDa EV-associated lipoprotein as the main source of the differential reactivity.

**Conclusions:**

LpqH could be a good plasma biomarker with capacity to distinguish PTB+ or MAP-vaccinated cows from cows infected with TB.

**Electronic supplementary material:**

The online version of this article (10.1186/s12917-019-1941-6) contains supplementary material, which is available to authorized users.

## Background

Both bovine tuberculosis (TB) and paratuberculosis (PTB) are serious and widespread bacterial infections affecting many domestic and wild animal species. *Mycobacterium avium subsp. paratuberculosis* (MAP) is the etiologic agent of paratuberculosis (PTB), a disease that causes severe granulomatous enteritis [1, 2]. Current data indicate that 50% of the European and North American bovine herds could be carrying MAP [3]. The damage associated to MAP infection includes economic losses due to the decrease in milk production by up to 10% or the culling of the infected animals 1. Among the control methods to fight against this devastating disease is vaccination [4]. However, current vaccines do not confer complete protection and cause interference with other diagnostics tests, including bovine TB. Therefore, the development of “Differentiating Infected from Vaccinated Animals” (DIVA) tests are a pressing need.

Mycobacteria, like many other intracellular bacteria, depend on specialized export systems to gain access to the extracellular milieu when they are confined inside the phagosome. In addition, many of these bacteria also release immunomodulatory factors via extracellular vesicles (EVs) [5, 6]. The production of EVs was described in Gram-negative bacteria more than 50 years ago and today it is acknowledged as a universal phenomenon observed in all domains of life. We have shown that both *M. tuberculosis* (Mtb) and *M. bovis* bacillus Calmmette-Guerin (BCG) release EVs in vitro and in vivo [7]. Moreover, we have described the first gene that seems to regulate vesiculogenesis in Mtb. A transposon mutant in *rv0431* showed an increased vesicle release and an attenuated phenotype in macrophage and murine infection models [8], suggesting a link between vesiculogenesis and mycobacterial pathogenesis. We had demonstrated that Mtb and BCG EVs are enriched in lipoproteins [7] that can reach the extracellular space independently of host-derived exosome production in Mtb-infected macrophages [9], suggesting that they could be circulating in the infected host as separate entities. This scenario suggests that mycobacterial EVs could represent good biomarkers for disease. In fact, we have also demonstrated that isolated Mtb EVs are recognized by sera from TB patients but not by tuberculin skin test positive (TST+) BCG-vaccinated control individuals [10]. Considering the limitation of current diagnostic tools in PTB and bovine TB+, we reasoned that it was worthwhile to investigate the biomarker potential of mycobacterial EVs in the contexts of these two diseases.

## Materials and methods

### Mycobacterial culture

*Mycobacterium bovis* strain NEIKER 1403 was originally isolated from a naturally infected wild boar at NEIKER*. M. avium subsp. paratuberculosis K10* and *M. tuberculosis* H37Rv strains were obtained from the ATCC. All strains were initially grown in Middlebrook 7H9 medium (M7H9) supplemented with 10% (v/v) OADC enrichment (Becton Dickinson Microbiology Systems, Spark, MD), 0.5% (v/v) glycerol and with or without Tyloxapol 0.05% (v/v; Sigma) prior to inoculation in a minimal medium (MM) consisting of KH_2_PO_4_ 1 g/l, Na_2_HPO_4_ 2.5 g/l, asparagine 0.5 g/l, ferric ammonium citrate 50 mg/l, MgSO_4_ × 7 H_2_O 0.5 g/l, CaCl_2_ 0.5 mg/l, ZnSO_4_ 0.1 mg/l, mycobactin J with or without Tyloxapol 0.05% (v/v), containing 0.1% (v/v) glycerol, pH 7.0. Cultures were grown for up to 14 d for Mtb and 4 weeks for *M. bovis* and MAP in 850 cm^2^ roller bottles (Corning) at 37 °C. All EVs preparations were obtained from MM culture supernatants. In some experiments, cultures were Mtb H37Rv cell lysates were generated as previously described [11].

### EV isolation and purification

EVs were isolated as described [7]. Briefly, mycobacterial cultures were pelleted at 3450 x*g* for 15 min, 4 °C and the supernatants were filtered through a 0.45-mm-pore size polyvinylidene difluoride filter (Millipore, Billerica, MA). The supernatants were then concentrated ~ 100-fold using an Amicon (Millipore) ultrafiltration system with a 100 kDa exclusion filter. The concentrate was then sequentially centrifuged at 4000 and 15,000 x*g* (15 min, 4 °C) to partially remove cell debris and aggregates, and the remaining supernatant was then centrifuged at 100,000 x*g* for 1 h at 4 °C to sediment the EV fraction. To obtain a pure EV preparation, the EV suspension was loaded onto an Optiprep (Sigma) density gradient as previously described [7, 12]. The fractions were then dialyzed separately in PBS overnight and recovered by sedimentation at 100,000 x*g* for 1 h. Finally, the vesicles were suspended in LPS-free PBS. Vesicle quantitation was performed using the BCA protein assay (Thermo Scientific, Rockford, IL) and the lipophilic probe DPH (1,6-Diphenyl-1,3,5-hexatriene-4′-trimethylammonium tosylate) (Invitrogen) as previously described [8].

### Expression and purification of recombinant LpqH

Mtb *lpqH* was amplified using *lpqH*F (5′-tgttcaagcaacaagtcga-3′) and *lpqH*R (5′-ttaggaacaggtcacctcg-3′) primers and cloned in pet23b (Novagen, using the *BamH*I and *Nde*I restriction sites to generate the lpqH::pet23b vector. *E. coli* BL21 (DE3) competent cells were transformed with lpqH::pet23b and cultures were grown in LB, induced with 0.1 mM IPTG at 23 °C for 16 h. Bacterial cells were harvested and lysed by sonication (30 s cycles, 6 times with 15 s resting periods) in lysis buffer (50 mM Tris-HCl pH 7.5, 150 mM NaCl, 10% glycerol and 10 mM imidazole. Lysed cells were centrifuged at 13,000 rpm for 30 min at 4 °C and the supernatants were passed through a Ni-NTA resin (Quiagen), washed in lysis buffer including 10 mM and 40 mM imidazole, and eluted in the same buffer including 250 mM imidazole. Fractions containing LpqH were pooled and concentrated using an Amicon Ultra filter tube (3 kDa) (Millipore). Concentrated fractions were then submitted to size exclusion chromatography using a Sephacryl S-200 column. Fractions containing LpqH were pooled and submitted to mass spectrometry analysis.

### Animals

Animals used in this study belonged to commercial farms and, towards collection of the data for this study, have been submitted only to the standard clinical practices specifically regulated by the European and Spanish legislation on tuberculosis and brucellosis control programs, plus fecal sampling.

Animals used in this study were submitted only to procedures that according to European (Directive 2010/63/EU of the European Parliament and of the Council of 22 September 2010 on the protection of animals used for scientific purposes. Chapter 1, Article 1, Section 5, paragraphs b and f) and Spanish (Real Decreto 53/2013, Article 2, Section 5, Paragraphs b and f) legislation on experimental animals are exempt from its application. The animals, belonging to registered farms supervised by the local livestock authority (Servicio de Ganadería de la Diputación Foral de Bizkaia, Servicio de Ganadería de la Diputación Forla de Gipuzkoa) were submitted only to the introduction of a needle in accordance with good veterinary practice and were not killed in relationship with this study.

Abattoirs were operated by two municipally owned companies and complied with the pertinent Basque (Basque Government Decree 454/1994), Spanish (Spanish Government Law 32/2007 and Royal decree 731/2007) and European (Council Regulation (EC) No 1099/2009) legislation on animal welfare, under the supervision of official veterinarians. Permission to collect and use the samples was obtained from the managers of each slaughterhouse: Matadero Municipal de Bilbao and Matadero Frigorífico Donostiarra S.A.L (MAFRIDO), respectively.

Non-infected cattle belonged to PTB/TB-free herds while MAP-vaccinated cattle came from TB-free herds included in a PTB control program with no MAP detection for the last 7 years. On the other hand, PTB+ animals were from a previous abattoir study [13, 14], whereas TB positive animals were selected for being comparative skin test reactors or from cattle with TB-compatible lesions detected at post mortem examination. All PTB+ and TB+ animals were MAP and *M. bovis* culture positive, respectively.

### Blood/plasma/serum samples

Bovine Plasma samples used in this study belonged to Friesian cattle grouped in 4 categories including mycobacteria non-infected cows, non-infected cows vaccinated (SILIRUM, CZ Veterinaria, Porriño, Spain) against PTB (MAP-vaccinated), MAP-infected animals (PTB+) and *M. bovis*-infected cattle (TB+). Mycobacterial infection was confirmed or ruled out by thorough pathological examination, culture, PCR, PTB indirect ELISA (IDvet, Grabels, France) and interferon-gamma release assay (IDvet). Blood was obtained from the caudal vein and collected in BD Vacutainer tubes (Becton, Dickinson and Company, Sparks, MD, USA). Plasma was separated by centrifugation at 1000 x*g* for 20 min and stored at − 20 °C until use. Murine serum samples were obtained from mice immunized with Mtb EVs as previously described [11].

### Elisa

Blood was obtained from the caudal vein and collected in BD Vacutainer tubes (Becton, Dickinson and Company, Sparks, MD, USA). Plasma was collected by centrifugation at 10000 x*g* for 20 min and stored at − 20 °C until use. Plasma reactivity was measured by ELISA against different sets of EVs, a whole cell lysate preparation of Mtb H37Rv or the purified Mtb lipoprotein LpqH. ELISA plates (96 wells) were coated with 20 μg/ml Mtb lysate, 10 μg/ml of EVs or 2 μg/ml of LpqH in PBS and incubated at 37 °C for 1 h and then blocked in PBS + 3% BSA for 1 h at 37 °C or overnight at 4 °C. Wells were washed three times in PBS with 0.05% Tween-20 (PBST) and then incubated with a 1:400 diluted cow plasma or mouse plasma for 1 h at 37 °C. After washing three times with PBST, the wells were incubated with a horseradish peroxidase (HRP)-conjugated goat antibody to bovine immunoglobulin G (Jackson Laboratories) or an HRP-conjugated mouse immunoglobulin G (Sigma) for 1 h at 37 °C. Color was developed with TMB (Sigma-Aldrich). The titer was defined as the dilution of sera resulting in an A_405_ 2 times greater than the background. Alternatively, sera were screened for the presence of antibodies against *M. bovis* with the IDEXX *M. bovis* Ab Test (Idexx Laboratories Inc.) following the instructions of the manufacturer.

### Immunoblot

Plasma reactivity against different antigenic preparations was also tested by immunoblotting [11, 15]. EVs from *M. bovis*, MAP or Mtb as well as a Mtb whole cell lysate or purified LpqH were separated by SDS-PAGE and transferred to nitrocellulose membranes, which were then blocked overnight in a buffer containing 5% milk in PBS with 0.1% Tween 20. Individual channels on a blotting frame were incubated with diluted bovine plasma (1:400) overnight at 4 °C. Channels were washed three times with PBS containing 0.1% Tween 20 and then incubated with an HRP-conjugated goat antibody to bovine immunoglobulin G (Sigma) for 1 h at RT. Membranes were developed using luminol (Pierce).

### Mass spectrometry analysis

Samples were loaded onto an SDS-PAGE gel in a buffer containing 50 mM Tris, pH 6.8, 5% glycerol, 1.67% β-mercaptoethanol, 1.67% SDS and 0.0062% bromophenol blue. Protein samples were boiled for 5 min and resolved in 12.5% acrylamide gels, using a Mini-Protean II electrophoresis cell (Bio-Rad). A constant voltage of 150 V was applied for 45 min. Gels were fixed stained with SimplyBlue Safestsain (Invitrogen) following the manufacturer’s instruction. The proteins of interest were cut and washed in milli-Q water. Reduction and alkylation were performed using ditiothreitol (10 mM DTT in 50 mM ammonium bicarbonate) at 56 °C for 20 min, followed by iodoacetamide (50 mM iodoacetamide in 50 mM ammonium bicarbonate) for another 20 min in the dark. Gel pieces were dried and incubated with trypsin (12.5 μg/ml in 50 mM ammonium bicarbonate) for 20 min on ice. After rehydration, the trypsin supernatant was discarded; Gel pieces were hydrated with 50 mM ammonium bicarbonate, and incubated overnight at 37 °C. After digestion, acidic peptides were cleaned with trifluoroacetic acid (TFA) 0.1% and dried out in a RVC2 25 speedvac concentrator (Christ). Peptides were resuspended in 10 μl 0.1% TFA and sonicated for 5 min prior to analysis. Peptide mixtures obtained from trypsin digestion were separated by online nanoLC and analyzed by electrospray tandem mass spectrometry. Peptide separation was performed on a nanoAcquity UPLC system (Waters) connected to an LTQ Orbitrap XL mass spectrometer (Thermo Electron). Samples were loaded onto a Symmetry 300 C18 UPLC Trap column, 180 μm × 20 mm, 5 μm (Waters). The precolumn was connected to a BEH130 C18 column, 75 μm × 200 mm, 1.7 μm (Waters) equilibrated in 3% acetonitrile and 0.1% FA, and peptides were eluted at 300 nl/min using a 30 min linear gradient of 3–50% acetonitrile directly onto the nanoelectrospray ion source (Proxeon Biosystems). The mass spectrometer automatically switched between MS and MS/MS acquisition in DDA mode. Survey full scan MS spectra (m/z 400–2000) were acquired in the orbitrap with a resolution of 30,000 at m/z 400. The 6 most intense ions were sequentially subjected to collision-induced dissociation (CID) fragmentation in the linear ion trap. Precursors with charge states of 2+ and 3+ were specifically selected for CID. Collision-energy applied to each peptide was automatically normalized as a function of the m/z and charge state. Analyzed peptides were excluded for further analysis during 30 s using dynamic exclusion list. Searches were performed using Mascot Search engine (Matrix Science) on Proteome Discoverer 1.2. software (Thermo Electron). Carbamidomethylation of cysteines as fixed modification and oxidation of methionines as variable modification. 10 ppm of peptide mass tolerance, and 0.5 Da fragment mass tolerance were adopted as search parameters. Spectra were searched against *Mycobacterium tuberculosis* H37Rv (as of 05 April 2017). A false discovery rate estimation procedure was applied for peptide identification, and only peptides passing FDR < 1% cutoff were considered as reliable hits.

### Cryo-electron microscopy (Cryo-EM)

EVs from *M. bovis* and MAP were fixed with 2% glutaraldehyde in 0.1 M cacodylate at room temperature for 2 h, and then incubated overnight in 4% formaldehyde, 1% glutaraldehyde, and 0.1% PBS. Grids were prepared following standard procedures and observed at liquid nitrogen temperatures in a JEM-2200FS/CR transmission electron microscope (JEOL Europe, Croissy-sur-Seine, France) operated at 200 kV. An in-column omega energy filter helped to record images with improved signal/noise ratio by zero-loss filtering. The energy selecting slit width was set at 9 eV. Digital images were recorded on an UltraScan4000 CCD camera under low-dose conditions at a magnification of 55,058 obtaining a final pixel size of 2.7 Å/pixel.

### Statistical analysis

Standard one-way ANOVA followed by a Tukey’s multiple comparison test of the means was used to determine statistical significance. A *p <* 0.05 was considered statistically significant.

## Results

### Characterization of *M. bovis* and MAP EVs

**Fig. 1:**
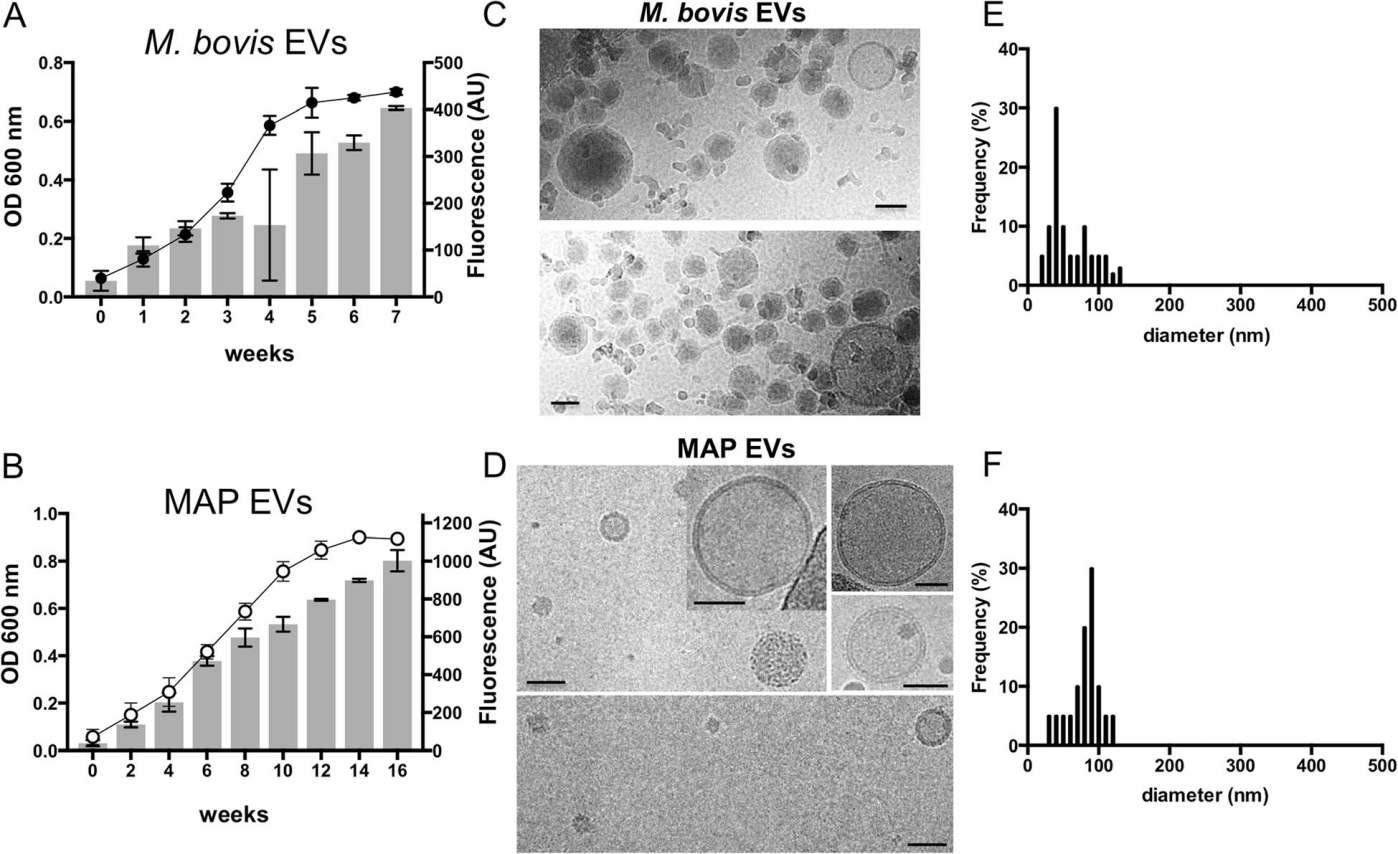
EVs from *Mycobacterium bovis* and *Mycobacterium avium paratuberculosis* (MAP). **a**, **b** Growth curves of *M. bovis* (**a**) and MAP (**b**) measured as OD_600_ nm. The release of EVs was analyzed by fluorescence measurements after treating clarified supernatants with DPH at the indicated time points. **c**, **d** EVs were isolated from cultures of *M. bovis* (**c**) and MAP (**d**) when cultures reached an OD = 0.6 and visualized by cryo-EM. Scale bars: 50 nm. All panels, including the insets represent independent fields of the indicated EV preparations. **e**, **f** The size distribution of EVs from *M. bovis* (**e**) and MAP (**f**) were determined by measuring the diameter of EVs in electron micrographs. Data are representative of 2 independent experiments

We have previously shown the conservation of EV production in the *Mycobacterium* genus [7]. Previous characterization of EVs was restricted to the most medically important species of mycobacteria for humans including *M. bovis* BCG and Mtb. Here we show in more detail isolated EVs from two of the most relevant mycobacterial species affecting both domestic animals and wildlife, including *M. bovis* and *M. avium* subsp. *paratuberculosis* (MAP). Since EV production is linked to cell division [16] and both *M. bovis* and especially MAP present longer generation times relative to Mtb we first assessed the kinetics of EVs production in these species. To investigate this, we measured the release of EVs at different time intervals in bacterial cells growing in MM using the lypophilic probe DPH [8, 17]. Using this methodology the release of EVs can be detected as early as day 5 in Mtb, corresponding to an OD_600_ nm of 0.1 (Additional file 1). As expected, EV production increased according to cell growth and it reached a plateau when bacterial cells entered the stationary phase. Similar behavior was observed in both *M. bovis* and MAP cultures (Fig. 1a, b). At a similar OD, Mtb and *M. bovis* produced more EVs than MAP according to the measured fluorescence. This is consistent with the increased generation time in MAP and confirms the relation of EV production and cell division. However, we cannot rule out that other factors could be affecting EVs, such as culture medium-specific restrictions are sensed differently by MAP and *M. bovis*. We performed an ultrastructural analysis of isolated EVs by transmission electron microscopy (TEM) (Fig. 1c, d). We could observe different EV morphologies in *M. bovis* EV preparations including bilayered vesicles and unilamelar vesicles (Fig. 1c). Conversely, most of the EVs in MAP preparations appeared as bilayered structures. These differences in morphology were accompanied by different size distributions. We observed a smaller mean diameter of *M. bovis* EVs (68 nm +/− 45 nm) compared to MAP (89 nm +/− 35 nm) (Fig. 1e, f). These results show that both *M. bovis* and MAP produce EVs with distinct kinetics, morphology and size.

### Immunogenicty of *M. bovis* and MAP EVs

**Fig. 2:**
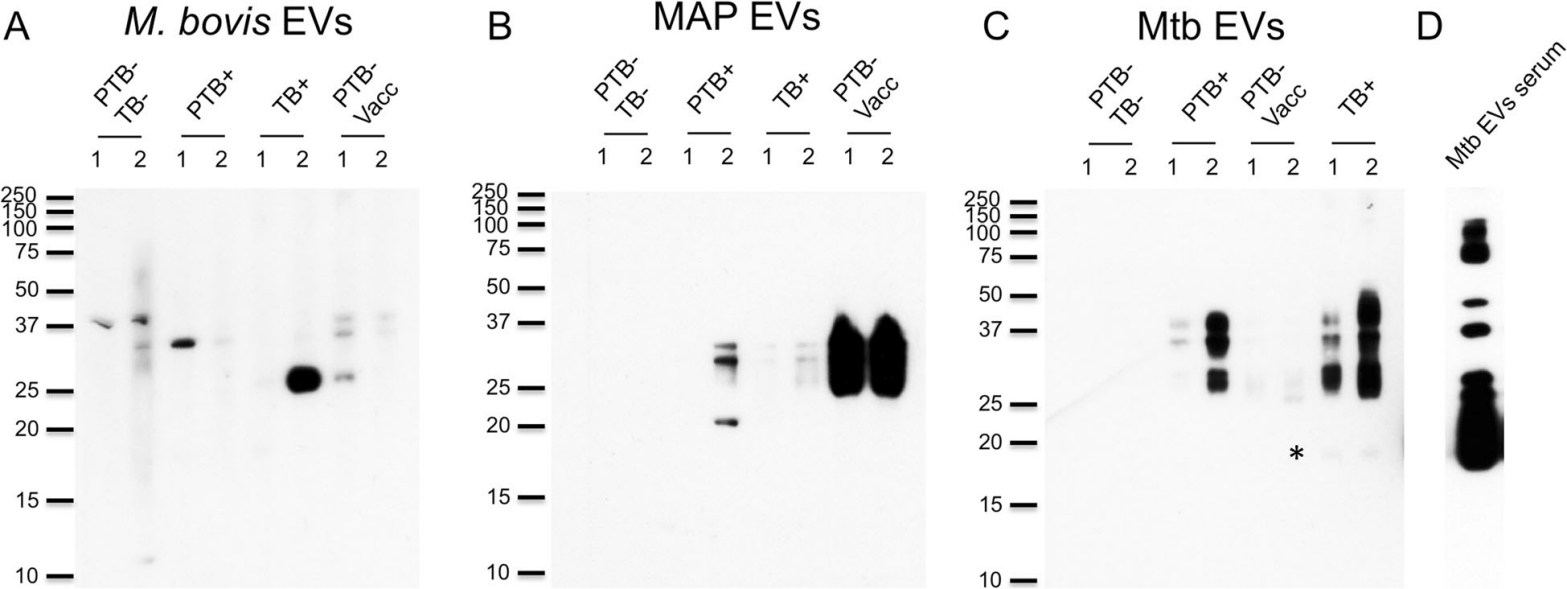
Immunogenicity of EVs from different mycobacterial strains. Isolated EVs from *M. bovis* (**a**), MAP (**b**) and Mtb H37Rv (**c**) were separated by SDS-PAGE and submitted to immunoblot with sera from two different cows grouped according to the following treatments: (1) (PTB- TB-), naïve cows; (2) (PTB+) Paratuberculosis positive; (3) (TB+) Bovine tuberculosis positive and; (4) (PTB- Vacc) PTB- and MAP vaccinated cows. **d** Mtb EVs were also submitted to immunoblot against Mtb EVs-specific serum. The asterisk indicates the presence of the 19 kDa antigen

The main goal of the latter study was to investigate whether Mtb EVs could represent a source of new TB serum biomarkers. Following the same rationale, we asked whether *M. bovis* and MAP EVs could have some discriminatory properties relative to each other. We determined the plasma reactivity of two independent samples from animals belonging to the following categories: (1) healthy (PTB−/TB-); (2) infected with MAP (PTB+); (3) infected with *M. bovis* (TB+) and; (4) healthy and vaccinated with heat-killed MAP (PTB−/Vacc.) (Fig. 2). We noticed some reactivity of 37 kDa and 35 kDa proteins in *M. bovis* EVs against sera from healthy cows. The latter protein was also reactive against sera from PTB+ cows. Of note, the highest reactivity was observed in one sample from TB+ cows to a protein of 26 kDa. In sera from MAP vaccinated cows, although it was noted to be more diverse, the reactivity was mostly associated with proteins of 26 kDa, 35 kDa and 36 kDa (Fig. 2a). When using EVs from MAP we observed no reactivity in healthy cows and some reactivity in PTB+ sera against three proteins of 20 kDa, 32 kDa and 35 kDa (Fig. 2b). Very low reactivity was observed in TB+ sera. The highest reactivity was observed in sera from MAP vaccinated animals, suggesting that part of the immunogenicity of the vaccine is due to some EVs-associated antigens (Fig. 2b). We also tested Mtb EVs and found no reactivity against sera from naïve cows and very reduced reactivity against sera from PTB−/ MAP vaccinated cows, (Fig. 2d). A similar pattern of reactivity between sera from PTB+ and TB+ cows was observed. However, we could notice that only TB+ sera recognized a 19 kDa protein, suggesting that Mtb EVs carry some antigens with capacity to discriminate PTB+ and PTB−/ MAP vaccinated cows from TB+ animals. As expected, an increase in the diversity of reactive Mtb EVs-associated proteins was measured when testing an Mtb EV immune serum (Fig. 2c, far right panel). We determined that half of the Mtb EV-associated proteins recognized by Mtb EV serum are recognized by PTB+ and TB+ sera (Fig. 2c). These results suggest that under the conditions tested Mtb EVs have some biomarker potential in plasma.

### Identification of reactive EV-associated proteins

We further proceeded to determine the identity of the Mtb EV-associated proteins recognized by TB+ sera. We run parallel immunoblot and coomassie SDS-PAGE and those bands reactive in the immunoblot were excised in the coomassie-stained gel and submitted to mass spectrometry (Table 1 and Additional file 2). We determined the identity of three reactive bands with masses of 30 kDa, 19 kDa and 7 kDa as conserved 35 kDa alanine-rich protein, lipoprotein LpqH and Acyl carrier protein AcpP, respectively. In a previous study we assessed Mtb EV immunogenicity by nucleic acid programmable protein array (NAPPA) and identified a total of 25 Mtb EV-associated reactive proteins against sera from Mtb-vaccinated mice [11]. The reduced number of reactive proteins found in the present study relative to the previous one, could be partially explained by the fact that the biology of EVs and the disease are different from that of human TB. In addition, the approach used in the present study provides a limited resolution and only proteins with the highest reactivity are selected.

**Table 1:** Mtb EV reactive proteins identified by mass spectrometry

Accession^a^	Protein Name	Score	Cov^b^	# Pept.
A2VJY2	Acyl carrier protein AcpP	390,08	44,35	5
A2VME2	19 kDa lipoprotein antigen lpqH	1157,9	11,95	2
A2VL81	Conserved 35 kDa alanine rich protein	980,9	61,48	6

^a^Accession number as Uniprop database

^b^Coverage as provided by Mascot mass spectrometry searching engine

### Discriminatory capacity of Mtb LpqH

**Fig. 3:**
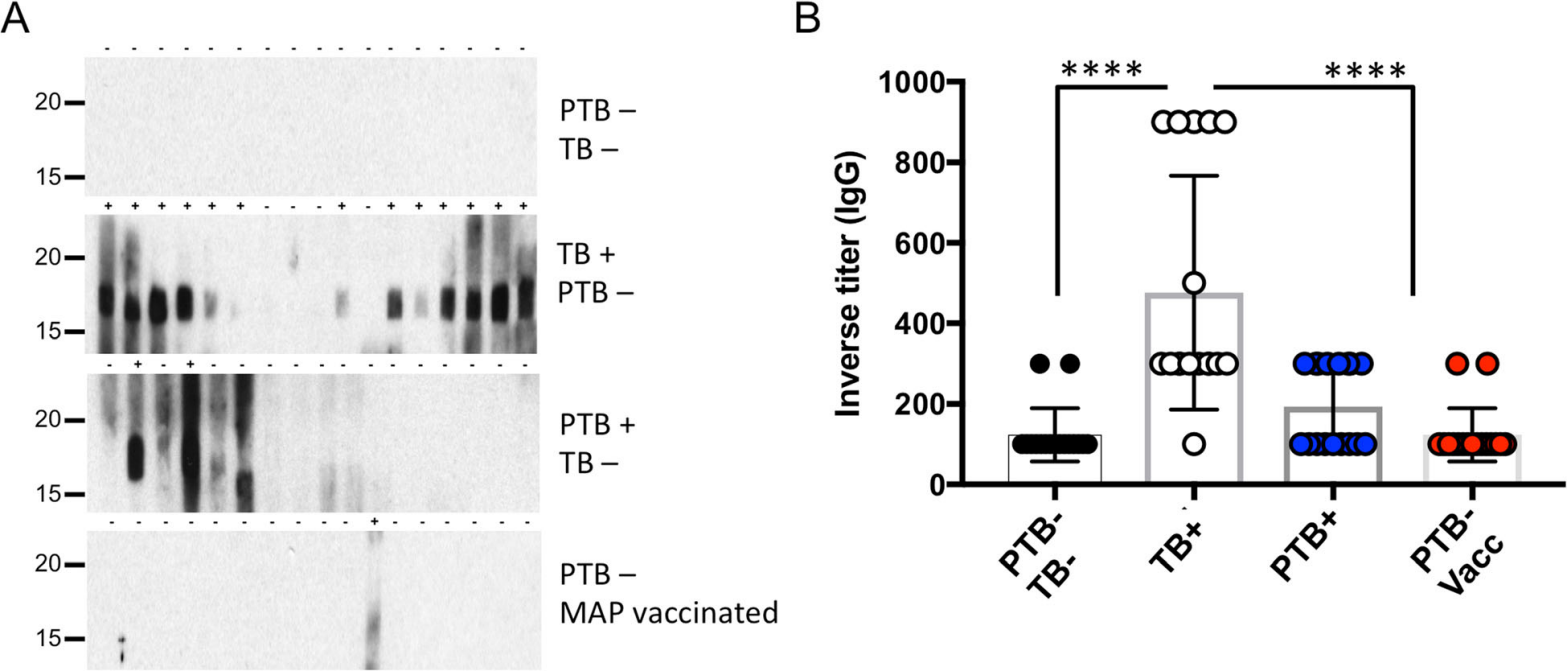
Reactivity of bovine sera to *M. tuberculosis* EV-associated lipoprotein LpqH. **a** Purified LpqH was separated by SDS-PAGE and submitted to immunoblot with sera from seventeen different cows grouped according to the following treatments: (1) (PTB- TB-), naïve cows; (2) (PTB+) Paratuberculosis positive; (3) (TB+) Bovine tuberculosis positive and; (4) (PTB- Vacc) PTB- and MAP vaccinated cows. Positive reactivity and absence of reactivity are indicated on top of each panel as “+” or “-”, respectively. **b** Inverse titers of LpqH-specific IgG antibodies measured by ELISA in the same seventeen bovine sera as in (**a**). Data are mean +/− sem. The results are representative of two independent and similar experiments. (**** *P* < 0.0001 one-way ANOVA with Tukey post-test)

We next tested whether Mtb LpqH could represent a good plasma biomarker. To do that, Mtb LpqH was recombinantly expressed in *E. coli* and subsequently purified by Ni-agarose affinity chromatography followed by size exclusion chromatography (Additional file 3A). Mass spectrometry analysis revealed that the band of 17 kDa observed in the SDS-PAGE of the pooled fractions is LpqH (Additional file 3B). We screened a set of 68 plasma samples pooled in 4 groups of 17 individual sera belonging to PTB-, PTB+, PTB- VAC and TB+ cows. The *M. bovis* infection status of each animal was previously confirmed by culture and histology and was assessed in this study using the IDEXX *M. bovis* Ab Test (Idexx Laboratories Inc.). No TB+ results were observed in the PTB- and PTB+ groups of samples. With this set of samples, we then tested LpqH as a biomarker. Immunoblot analysis showed no reactivity in the PTB- TB- group (Fig. 3a). Only, two out of 17 samples (12%) reacted against the 19 kDa LpqH antigen in the PTB+ group. Importantly, no reactivity against LpqH was measured in sera from PTB−/MAP-vaccinated cows. Conversely, 13 out of 17 sera (77%) from TB+ cows contained LpqH-reactive antibodies (Fig. 3a). These results were confirmed by ELISA by measuring LpqH-reactive bovine total IgGs. The highest LpqH-specific IgG titers were measured in sera from TB+ cows with statistically significant differences to the other groups (Fig. 3b). Taking together, these results suggest that Mtb LpqH represents a good biomarker to differentiate between PTB+ or PTB- VAC animals from TB+ cows.

## Discussion

The goal of this study was to investigate the feasibility of EVs as a source of plasma biomarkers in the context of PTB and bovine TB. The characterization of EVs from MAP and *M. bovis* showed that, although EV production is conserved, their kinetics and morphology differ between these two species, suggesting a divergent biological significance. These results might also reflect the unique underlying physiology of each pathogen and encourage investigating the specific requirements for EV production in more detail. We have previously demonstrated that Mtb EVs are immunogenic in HIV negative patients with pulmonary TB [10]. More specifically, we showed that a mix of three EV-associated proteins of 36, 25 and 23 kDa could react against serum from smear-positive and smear-negative TB patients but not against skin test positive, BCG vaccinated individuals, suggesting the diagnostic potential of EVs in TB. In a different study, we showed that Mtb EVs are immunogenic and provide protection in mice similar to that observed with BCG, when administered prior to an aerosol challenge with virulent Mtb [11]. In the later study, we demonstrated that a few EV-associated proteins explained the immunogenicity of Mtb’s EVs. When studying the immunogenicity of MAP and *M. bovis* EVs we noticed that both are cross-reactive against independent plasma samples from cows that had been infected with MAP, vaccinated with inactivated MAP or infected with *M. bovis*. Even *M. bovis* EVs were reactive against sera from healthy cows, which make these EVs unsuitable as source of biomarkers. Although MAP EVs did not react against healthy sera, they were recognized similarly by sera from PTB+ cows, vaccinated cows and TB+ cows. These results indicate that EVs from neither MAP nor *M. bovis* could be used as biomarkers in the conditions tested. Conversely, we found that Mtb EVs are reactive against TB+ sera and PTB+ sera but not against sera from MAP-vaccinated cows. In this regard, we noticed a differential recognition of Mtb EV-associated LpqH by sera from TB+ cows relative to that of PTB+, suggesting that these EVs do have some diagnostic potential. In our previous study on Mtb EVs as source of TB biomarkers in human serum, we noticed that a significant portion of the immunogenicity of Mtb EVs is associated to lipoarabinomannan (LAM), the major cell wall-associated lipopolysaccharide in Mtb [10]. Interestingly, this effect was not observed in BCG EVs, suggesting differences in either the organization of LAM on EVs or some antigenic variability associated to the polysaccharide. We believe that the fact that we could not measure an expected reactivity of Mtb EVs against MAP-vaccinated sera might be partially explained by the way LAM is presented via vaccination that should be different to that of the association of LAM to EVs. These differences might have been introduced during the heat inactivation process of MAP for vaccine preparation.

The conventional kits for PTB disease are based on ELISA and while they provide a high specificity (90–99%) they show low sensitivity (13–40%) [18]. It has been reported that a strong correlation exists between the amount of MAP in the feces and the sensitivity obtained by these commercial kits [19]. One recent study demonstrated that a different antigenic composition from the one included in the commercial ELISA, based on secreted antigens could bypass the dependence of the sensitivity of the kit on the fecal shedding [20]. EVs are released cellular products with a unique composition relative to the cell. Although we could not demonstrate that either *M. bovis* or MAP EVs are good sources of biomarkers we believe that besides protein antigens, other biomolecules associated to EVs could have some diagnostic potential, including the LAM-related polysaccharides. In addition, different growth media could influence EV production in these species. Proteomic studies of both MAP and *M.bovis* EVs should offer some valuable information about the similarity between EVs cargo among mycobacterial species and will help to explain the observed differences in reactivity. All these findings are in agreement with the observed complex immunopathological dynamics of mycobacterial diseases. Understanding how these mycobacterial related species release EVs will be relevant to assess the potential of these biomolecules as source of biomarkers.

## Conclusions

The results shown here indicate that mycobacterial EVs may represent a novel source of biomarkers. Characterization of EV production in both Map and *M. bovis* demonstrates that this process might be dictated by a different physiology and encourage further studies on this process. A single EV-associated protein can discriminate between PTB and TB in cows, what points to a novel DIVA biomarker.

## Additional files

Additional file 1: Kinetic of EV production by *M. tuberculosis* H37Rv. Growth curve of Mtb H37Rv in MM (solid dark line). EV production by Mtb as measured by using the fluorescence probe DPH (grey bars). Data represent the mean +/− standard error. The data shown are representative of two independent experiments. (TIF 1521 kb)

Additional file 2: Identification of bovine plasma most reactive proteins to Mtb EVs. Mtb EVs were separated by SDS-PAGE and blotted onto a nitrocellulose membrane. Plasma from a TB + cow was used to study the subset of proteins present in EVs that are able to raise antibodies. Several reactive proteins were identified. Details on mass spectrometry identifications are indicated in Table 1. (TIF 1521 kb)

Additional file 3: Purification of recombinant Mtb LpqH. (A) SDS-PAGE of fractions of a size exclusion chromatography of eluted His_6_-LpqH. Fractions 27–30 were pooled submitted to mass spectrometry analysis. (B) Mascot result snapshot of the band including recombinant LpqH. (TIF 1521 kb)

## Data Availability

All data generated or analysed during this study will be available from the corresponding author on reasonable request,

## Abbreviations

EV: Extracelular vesicle
MAP: *Mycobacterium avium subsp. paratuberculosis*
MS: Mass spectrometry
Mtb: *Mycobacterium tuberculosis*
PTB: Paratuberculosis
TB: Bovine tuberculosis

## References

[1] Eslami M, Shafiei M, Ghasemian A, Valizadeh S, Al-Marzoqi AH, Shokouhi Mostafavi SK, Nojoomi F, Mirforughi SA (2019). Mycobacterium avium paratuberculosis and Mycobacterium avium complex and related subspecies as causative agents of zoonotic and occupational diseases. J Cell Physiol.

[2] Sweeney RW (2011). Pathogenesis of paratuberculosis. Vet Clin North Am Food Anim Pract.

[3] McAloon CG, Roche S, Ritter C, Barkema HW, Whyte P, More SJ, O'Grady L, Green MJ, Doherty ML (2019). A review of paratuberculosis in dairy herds - part 2: on-farm control. Vet J.

[4] Alonso-Hearn M, Molina E, Geijo M, Vazquez P, Sevilla IA, Garrido JM, Juste RA (2012). Immunization of adult dairy cattle with a new heat-killed vaccine is associated with longer productive life prior to cows being sent to slaughter with suspected paratuberculosis. J Dairy Sci.

[5] Schwechheimer C, Kuehn MJ (2015). Outer-membrane vesicles from gram-negative bacteria: biogenesis and functions. Nat Rev Microbiol.

[6] Brown L, Wolf JM, Prados-Rosales R, Casadevall A (2015). Through the wall: extracellular vesicles in gram-positive bacteria, mycobacteria and fungi. Nat Rev Microbiol.

[7] Prados-Rosales R, Baena A, Martinez LR, Luque-Garcia J, Kalscheuer R, Veeraraghavan U, Camara C, Nosanchuk JD, Besra GS, Chen B (2011). Mycobacteria release active membrane vesicles that modulate immune responses in a TLR2-dependent manner in mice. J Clin Invest.

[8] Rath P, Huang C, Wang T, Wang T, Li H, Prados-Rosales R, Elemento O, Casadevall A, Nathan CF (2013). Genetic regulation of vesiculogenesis and immunomodulation in Mycobacterium tuberculosis. Proc Natl Acad Sci U S A.

[9] Athman JJ, Wang Y, McDonald DJ, Boom WH, Harding CV, Wearsch PA (2015). Bacterial membrane vesicles mediate the release of Mycobacterium tuberculosis Lipoglycans and lipoproteins from infected macrophages. J Immunol.

[10] Ziegenbalg A, Prados-Rosales R, Jenny-Avital ER, Kim RS, Casadevall A, Achkar JM (2013). Immunogenicity of mycobacterial vesicles in humans: identification of a new tuberculosis antibody biomarker. Tuberculosis (Edinb).

[11] Prados-Rosales R, Carreno LJ, Batista-Gonzalez A, Baena A, Venkataswamy MM, Xu J, Yu X, Wallstrom G, Magee DM, LaBaer J (2014). Mycobacterial membrane vesicles administered systemically in mice induce a protective immune response to surface compartments of Mycobacterium tuberculosis. MBio.

[12] Prados-Rosales R, Brown L, Casadevall A, Montalvo-Quiros S, Luque-Garcia JL (2014). Isolation and identification of membrane vesicle-associated proteins in gram-positive bacteria and mycobacteria. MethodsX.

[13] Vazquez P, Garrido JM, Juste RA (2013). Specific antibody and interferon-gamma responses associated with immunopathological forms of bovine paratuberculosis in slaughtered Friesian cattle. PLoS One.

[14] Vazquez P, Ruiz-Larranaga O, Garrido JM, Iriondo M, Manzano C, Agirre M, Estonba A, Juste RA (2014). Genetic association analysis of paratuberculosis forms in Holstein-friesian cattle. Vet Med Int.

[15] Saha DC, Goldman DL, Shao X, Casadevall A, Husain S, Limaye AP, Lyon M, Somani J, Pursell K, Pruett TL (2007). Serologic evidence for reactivation of cryptococcosis in solid-organ transplant recipients. Clin Vaccine Immunol.

[16] Kulp A, Kuehn MJ (2010). Biological functions and biogenesis of secreted bacterial outer membrane vesicles. Annu Rev Microbiol.

[17] Crowley JT, Toledo AM, LaRocca TJ, Coleman JL, London E, Benach JL (2013). Lipid exchange between Borrelia burgdorferi and host cells. PLoS Pathog.

[18] Sweeney RW, Whitlock RH, McAdams S, Fyock T (2006). Longitudinal study of ELISA seroreactivity to Mycobacterium avium subsp. paratuberculosis in infected cattle and culture-negative herd mates. J Vet Diagn Investig.

[19] Clark DL, Koziczkowski JJ, Radcliff RP, Carlson RA, Ellingson JL (2008). Detection of Mycobacterium avium subspecies paratuberculosis: comparing fecal culture versus serum enzyme-linked immunosorbent assay and direct fecal polymerase chain reaction. J Dairy Sci.

[20] Facciuolo A, Kelton DF, Mutharia LM (2013). Novel secreted antigens of Mycobacterium paratuberculosis as serodiagnostic biomarkers for Johne's disease in cattle. Clin Vaccine Immunol.

