# Supplementary figures and images for: Mycobacterium tuberculosis extracellular vesicle-associated lipoprotein LpqH as a potential biomarker to distinguish paratuberculosis infection or vaccination from tuberculosis infection
Source: BMC Vet Res. 2019 Jun 7;15:188. doi: 10.1186/s12917-019-1941-6 (PMC6555097; doi:10.1186/s12917-019-1941-6)

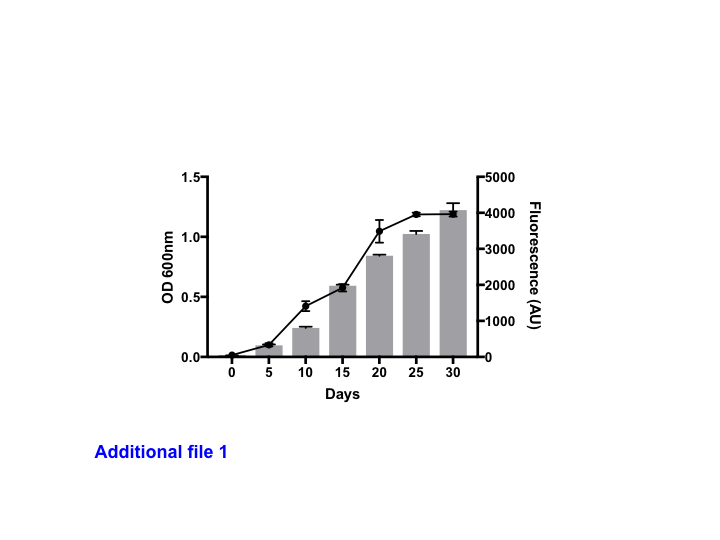

Supplement: Supplementary file 1 — Kinetic of EV production by M. tuberculosis H37Rv. Growth curve of Mtb H37Rv in MM (solid dark line). EV production by Mtb as measured by using the fluorescence probe DPH (grey bars). Data represent the mean +/− standard error. The data shown are representative of two independent experiments. (TIF 1521 kb) [file 12917_2019_1941_MOESM1_ESM.tif]

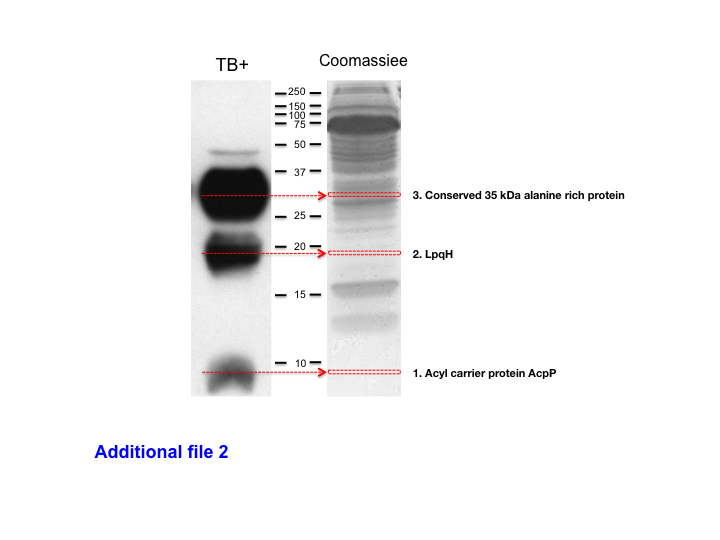

Supplement: Supplementary file 2 — Identification of bovine plasma most reactive proteins to Mtb EVs. Mtb EVs were separated by SDS-PAGE and blotted onto a nitrocellulose membrane. Plasma from a TB + cow was used to study the subset of proteins present in EVs that are able to raise antibodies. Several reactive proteins were identified. Details on mass spectrometry identifications are indicated in Table 1. (TIF 1521 kb) [file 12917_2019_1941_MOESM2_ESM.tif]

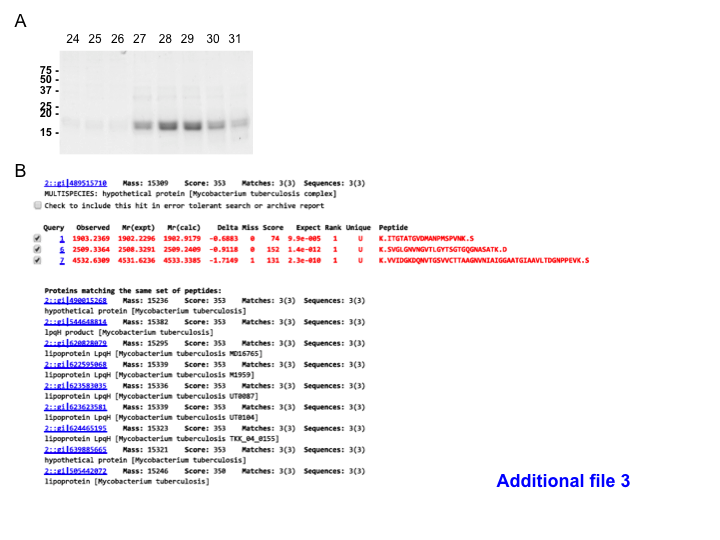

Supplement: Supplementary file 3 — Purification of recombinant Mtb LpqH. (A) SDS-PAGE of fractions of a size exclusion chromatography of eluted His6-LpqH. Fractions 27–30 were pooled submitted to mass spectrometry analysis. (B) Mascot result snapshot of the band including recombinant LpqH. (TIF 1521 kb) [file 12917_2019_1941_MOESM3_ESM.tif]
